# *De novo* mutations in children born after medical assisted reproduction

**DOI:** 10.1093/humrep/deac068

**Published:** 2022-04-12

**Authors:** R M Smits, M J Xavier, M S Oud, G D N Astuti, A M Meijerink, P F de Vries, G S Holt, B K S Alobaidi, L E Batty, G Khazeeva, K Sablauskas, L E L M Vissers, C Gilissen, K Fleischer, D D M Braat, L Ramos, J A Veltman

**Affiliations:** Department of Obstetrics and Gynaecology, Radboudumc, Nijmegen, the Netherlands; Biosciences Institute, Faculty of Medical Sciences, Newcastle University, Newcastle upon Tyne, UK; Department of Human Genetics, Donders Institute for Brain, Cognition and Behaviour, Radboudumc, Nijmegen, the Netherlands; Department of Human Genetics, Donders Institute for Brain, Cognition and Behaviour, Radboudumc, Nijmegen, the Netherlands; Department of Obstetrics and Gynaecology, Radboudumc, Nijmegen, the Netherlands; Department of Human Genetics, Donders Institute for Brain, Cognition and Behaviour, Radboudumc, Nijmegen, the Netherlands; Biosciences Institute, Faculty of Medical Sciences, Newcastle University, Newcastle upon Tyne, UK; Biosciences Institute, Faculty of Medical Sciences, Newcastle University, Newcastle upon Tyne, UK; Biosciences Institute, Faculty of Medical Sciences, Newcastle University, Newcastle upon Tyne, UK; Department of Human Genetics, Radboud Institute for Molecular Life Sciences, Radboudumc, Nijmegen, the Netherlands; Department of Human Genetics, Radboud Institute for Molecular Life Sciences, Radboudumc, Nijmegen, the Netherlands; Department of Human Genetics, Donders Institute for Brain, Cognition and Behaviour, Radboudumc, Nijmegen, the Netherlands; Department of Human Genetics, Radboud Institute for Molecular Life Sciences, Radboudumc, Nijmegen, the Netherlands; Department of Obstetrics and Gynaecology, Radboudumc, Nijmegen, the Netherlands; Department of Obstetrics and Gynaecology, Radboudumc, Nijmegen, the Netherlands; Department of Obstetrics and Gynaecology, Radboudumc, Nijmegen, the Netherlands; Biosciences Institute, Faculty of Medical Sciences, Newcastle University, Newcastle upon Tyne, UK

**Keywords:** infertility, medical assisted reproduction, *de novo* mutations, whole-genome sequencing, intracytoplasmic sperm injection, paternal age

## Abstract

**STUDY QUESTION:**

Are there more *de novo* mutations (DNMs) present in the genomes of children born through medical assisted reproduction (MAR) compared to spontaneously conceived children?

**SUMMARY ANSWER:**

In this pilot study, no statistically significant difference was observed in the number of DNMs observed in the genomes of MAR children versus spontaneously conceived children.

**WHAT IS KNOWN ALREADY:**

DNMs are known to play a major role in sporadic disorders with reduced fitness such as severe developmental disorders, including intellectual disability and epilepsy. Advanced paternal age is known to place offspring at increased disease risk, amongst others by increasing the number of DNMs in their genome. There are very few studies reporting on the effect of MAR on the number of DNMs in the offspring, especially when male infertility is known to be affecting the potential fathers. With delayed parenthood an ongoing epidemiological trend in the 21st century, there are more children born from fathers of advanced age and more children born through MAR every day.

**STUDY DESIGN, SIZE, DURATION:**

This observational pilot study was conducted from January 2015 to March 2019 in the tertiary care centre at Radboud University Medical Center. We included a total of 53 children and their respective parents, forming 49 trios (mother, father and child) and two quartets (mother, father and two siblings). One group of children was born after spontaneous conception (n = 18); a second group of children born after IVF (n = 17) and a third group of children born after ICSI combined with testicular sperm extraction (ICSI-TESE) (n = 18). In this pilot study, we also subdivided each group by paternal age, resulting in a subgroup of children born to younger fathers (<35 years of age at conception) and older fathers (>45 years of age at conception).

**PARTICIPANTS/MATERIALS, SETTING, METHODS:**

Whole-genome sequencing (WGS) was performed on all parent-offspring trios to identify DNMs. For 34 of 53 trios/quartets, WGS was performed twice to independently detect and validate the presence of DNMs. Quality of WGS-based DNM calling was independently assessed by targeted Sanger sequencing.

**MAIN RESULTS AND THE ROLE OF CHANCE:**

No significant differences were observed in the number of DNMs per child for the different methods of conception, independent of parental age at conception (multi-factorial ANOVA, *f*(2) = 0.17, *P*-value = 0.85). As expected, a clear paternal age effect was observed after adjusting for method of conception and maternal age at conception (multiple regression model, *t* = 5.636, *P*-value = 8.97 × 10^−7^), with on average 71 DNMs in the genomes of children born to young fathers (<35 years of age) and an average of 94 DNMs in the genomes of children born to older fathers (>45 years of age).

**LIMITATIONS, REASONS FOR CAUTION:**

This is a pilot study and other small-scale studies have recently reported contrasting results. Larger unbiased studies are required to confirm or falsify these results.

**WIDER IMPLICATIONS OF THE FINDINGS:**

This pilot study did not show an effect for the method of conception on the number of DNMs per genome in offspring. Given the role that DNMs play in disease risk, this negative result is good news for IVF and ICSI-TESE born children, if replicated in a larger cohort.

**STUDY FUNDING/COMPETING INTEREST(S):**

This research was funded by the Netherlands Organisation for Scientific Research (918-15-667) and by an Investigator Award in Science from the Wellcome Trust (209451). The authors have no conflicts of interest to declare.

**TRIAL REGISTRATION NUMBER:**

N/A.

## Introduction

One of the main revolutions in the area of medical assisted reproduction (MAR) was the introduction of ICSI in 1992 for the treatment of male infertility (Palermo *et al.*, 1992), and shortly after that, the combined use of sperm retrieved with testicular sperm extraction for azoospermia cases (Silber *et al.*, 1995). Sperm of low quality or quantity that was previously unlikely to fertilize via *in vivo* fertilization or IVF could now be used to fertilize oocytes and produce offspring. In the last decade, the use of ICSI has intensified from 36.4% in 1995 to over 76% in 2012 (Li *et al.*, 2018) and it is now routinely applied in more extreme cases of spermatogenic failure and between 68% and 72% in cases with (non-male factor) indications such as fertilisation failure (Glenn *et al.*, 2021).

Several studies have investigated the long-term health risks of ICSI children and showed no association with major congenital abnormalities (Woldringh *et al.*, 2008, 2010; Catford *et al.*, 2018; Pastuszak *et al.*, 2019). However, large epidemiological studies showed increased risks of lower birthweight, preterm delivery, minor congenital anomalies and rare imprinting disorders (Bonduelle *et al.*, 2002, 2004; Wisborg *et al.*, 2010; Belva *et al.*, 2011; Lazaraviciute *et al.*, 2014). Inconsistencies remain regarding the increased risks of impaired cognitive development, neurodevelopmental disorders, metabolic health and the effect on reproductive fitness (Woldringh *et al.*, 2008, 2010; Catford *et al.*, 2018; Rumbold *et al.*, 2019; Briana and Malamitsi-Puchner, 2022). Even if these health risks are real, it remains unclear whether they are caused by the ART employed in sperm retrieval, ovarian stimulation, method of conception or laboratory conditions, are caused by the underlying parental factors or can be exacerbated due to bypassing natural selection.

While most of our DNA is inherited without any changes, copying mistakes during DNA replication result in *de novo* mutations (DNMs) arising in the germline of each new-born. On average, a human genome contains 44 to 89 *de novo* single nucleotide variations (Kong *et al.*, 2012; Francioli *et al.*, 2015; Acuna-Hidalgo *et al.*, 2016; Goldmann *et al.*, 2016). While these numbers are relatively small compared to the millions of inherited genetic variations present in our genomes, it has become clear that DNMs are a major cause of severe early-onset disease such as intellectual disability (Veltman and Brunner, 2012; Gilissen *et al.*, 2014; Taylor *et al.*, 2019). A risk factor for the increase of DNMs in offspring is parental age at the time of conception, especially paternal age. Approximately 1.35 to 1.5 DNMs are added to the germline of offspring with each additional paternal year (Li *et al.*, 1996; Crow, 2000; Kong *et al.*, 2012; Jónsson *et al.*, 2017; Kessler *et al.*, 2020). In comparison, the number of DNMs with increasing maternal age is much lower with only an extra 0.24 to 0.42 DNM with each additional maternal year (Acuna-Hidalgo *et al.*, 2016; Goldmann *et al.*, 2016; Jónsson *et al.*, 2017; Kessler *et al.*, 2020). So far, no large-scale studies have looked in depth at the impact of MAR on the number of DNMs in the offspring. Interestingly, however, Wong *et al.* (2016) mentioned that the usage of MAR to achieve conception resulted in 4.25 more DNMs on average per genome compared with natural conception when controlling for other variables, a result described as moderately significant (Wong *et al.*, 2016). Unfortunately, the authors did not discuss these results any further, in fact they excluded the data from these 25 children born through MAR from further analyses. A more recent study also indicated that children born through MAR carried more DNMs in their genome than naturally conceived children (Wang *et al.*, 2021). In this study, we wanted to further elaborate on this work and study whether indeed children conceived using IVF and ICSI combined with testicular sperm extraction (ICSI-TESE) are more likely to have a higher number of DNMs in their genome than children born after spontaneous conception. In order to determine whether a potential effect may be linked to the paternal age at conception, we decided to include children born to young fathers (<35 years of age at time of conception) as well as children born to older fathers (>45 years of age at time of conception).

## Materials and methods

### Ethical approval

The study was approved by the Committee on Research Involving Human Subjects (CMO) in Nijmegen, the Netherlands on 28 August 2014 (file number NL49308.091.14). Written informed consent was obtained from each family and signed by both parents. Informed consent only entailed the analyses of the number of DNMs, no further analysis regarding the pathogenicity or clinical implications of these DNMs was allowed.

### Selection criteria and population

This observational study was conducted from January 2015 to March 2019 at the Department of Obstetrics and Gynaecology and the department of Human Genetics at the tertiary care centre of the Radboud University Medical Center (Radboudumc). Couples included in this study were recruited from couples referred to the Radboudumc fertility department between 2003 and 2017 with fertility problems, predominantly male factor infertility, which successfully conceived and resulted in a live birth after either IVF or ICSI-TESE. Couples where the man suffered from azoospermia (no sperm in ejaculate) or extreme oligozoospermia (<0.1 million sperm/ml) were recommended the ICSI-TESE treatment. Couples that spontaneously conceived and delivered a baby at the Radboudumc during this period were asked to participate in the study’s control group, even if the father had been previously diagnosed with male factor infertility.

Exclusion criteria to participate in this study were a severe language barrier prohibiting a complete understanding of the consent form, inability of one of the subjects (parents or children) to participate in saliva sampling, presence of known chromosomal aberrations in father such as azoospermia factor deletions, children born after donation of oocytes or semen, children born to fathers who had chemotherapy or radiation therapy due to a malignancy and children born to couples who had IVF treatment solely due to male infertility. Eligible candidate trios (mother, father and child) received written study information followed by a telephone consultation approximately 2 weeks later. After written informed consent was obtained, DNA collection kits (Oragene·DNA OG-500^®^, DNA Genotek Inc., Ottawa, Canada) were sent to the family for the collection and return of saliva samples together with a short questionnaire requesting information regarding the baseline characteristics of each individual. This self-reported information (Supplementary Table SI) was later used for the identification of potential risk factors in relation to the occurrence of DNMs in offspring.

**Figure 1.**
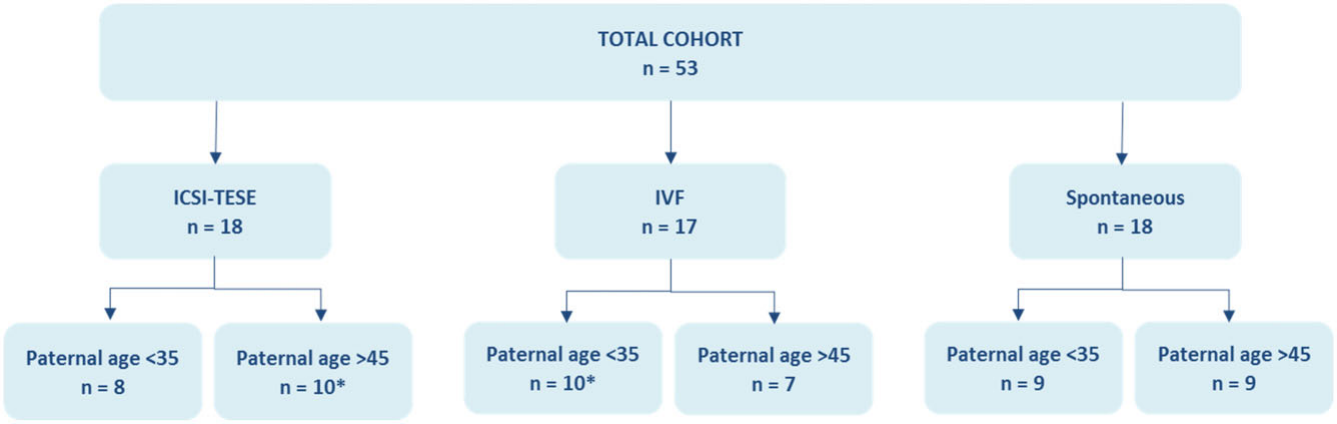
**Schematic presentation of the study cohort.** Families were divided into three groups according to the method of conception, either conceived spontaneous or via medical assisted reproduction (IVF or ICSI combined with testicular sperm extraction (ICSI-TESE)). Families were further divided into those born to young fathers, those younger than 35 years of age at the time of conception (<35), or older fathers, those older than 45 years of age years of age at conception (>45). Groups marked with * include one family composed by mother, father and two siblings.

A total of 53 children and their respective parents composing 49 trios (mother, father and child) and 2 quartets (mother, father and two siblings) were included in this study. Children were divided into three groups: children born after spontaneous conception (n = 18), children born after IVF (n = 17) and children born after ICSI-TESE (n = 18). In order to investigate the influence of paternal age at time of conception on the method of conception, the three groups (spontaneous, IVF and ICSI-TESE) were subdivided accordingly into two groups, one with young fathers (<35 years of age at time of conception) and another with older fathers (>45 years at time of conception). At the time of selection, the children were between 1 and 10 years old. The detailed scheme of the study cohort is represented in Fig. 1.

All fathers in the ICSI-TESE group with age >45 years at time of conception were known to have secondary infertility due to having had a vasectomy in the past and proven fertility by having spontaneously conceived children in previous relationships. As for the two quartets, the siblings from one family were both conceived via IVF but during separate treatment cycles; while the siblings from the other family were dizygotic twins conceived simultaneously following the same ICSI-TESE procedure. Information obtained after the inclusion of couples in the control group showed that some of them had conceived spontaneously (n = 6) after having previously received a consultation at the Fertility department following a lack of conception after 12 months of unprotected intercourse. Since these couples managed to conceive spontaneously after a period of expectative management or while waiting for a fertility treatment, they were included and counted among the controls.

### Whole-genome sequencing, quality control and validation

Genomic DNA was isolated from all saliva samples with chemagic STAR DNA Saliva4k (PerkinElmer, England, UK) following the manufacturer’s instructions. The genomes of the 149 individuals were prepared using the DNA Library Prep Kit (BGI, MA, USA) following the manufacturer’s protocol. Genome sequencing was performed on the BGIseq-500 sequencer at Beijing Genomics Institute (BGI) at an average depth of 38× per sample (Supplementary Table SII). Sequenced reads were aligned to the Human Reference Genome (GRCh37.p5/hg19) using BWA-Mem (Li and Durbin, 2010), single-nucleotide variations and small indels were identified and quality-filtered using xAtlas (Farek *et al.*, 2018). DNMs were independently called using the standard in-house algorithm and using DeNovoCNN, a deep-learning custom-designed algorithm (Khazeeva *et al.*, 2021). DNMs called by both methods with a minimum of 10 reads carrying the mutation were deemed high-confidence DMNs. For 102 out of 149 individuals (68%), whole-genome sequencing (WGS) was performed twice. For these samples DNMs identified in both experiments were considered true DNMs. Sanger sequencing was used to validate that called DNMs were real and only present in the children.

### Phasing analysis and determining parent-of-origin

The allelic and parent of origin analysis of all DNMs found in the autosomes of each child was performed using parentally informative single-nucleotide polymorphisms (iSNPs), i.e. individual single-nucleotide polymorphism that could conclusively be traced back to a single parent. Phasing was performed on DNMs, where iSNPs were within a range of 5000 base pairs (bp) to either side of the DNM, using an in-house pipeline that incorporates WhatsHap (Patterson *et al.*, 2015; Martin *et al.*, 2016), bamql (Masella *et al.*, 2016) and SAMtools (Li *et al.*, 2009) to perform fast target phasing for each DNM-iSNP region. Where possible, using overlapping reads across variants allowed regions larger than the standard 150 bp read lengths to be phased, with the majority of phasable DNMs having an iSNP within less of 800 bp from it.

### Statistical analysis

Descriptive statistics were used to calculate the number of DNMs per child and treatment. Power analysis calculations were performed to determine the minimum effect sizes that could be detected in this study based on experimental design. A multi-factorial ANOVA test was used to determine the effect of treatment and parental age on the number of DNMs in the genome of all the children with pairwise comparisons between groups performed *post hoc* via Tukey test using the Bonferroni method to correct error rates when performing multiple tests. Multiple regression models were used to adjust for paternal, maternal and paternal age when determining the effect of method of conception in the number of DNMs found in children and determine whether a single variable on its own or a combination of variable were responsible for the increase in number of DNMs identified in the children. Differences in the proportion of DNMs originating in the paternal alleles or maternal alleles between groups were analysed using multi-factorial ANOVA tests. All statistical analyses were performed using R statistical software (R Core Team, 2020).

### Gene ontology enrichment analysis

The 82 DNMs found in the previous analysis to affect the coding region of the genome of the children in each group were extracted and further investigated. Gene ontology (GO) terms and enrichment analysis between each group were performed using Bioconductor’s topGO package in R (R Core Team, 2020; Alexa and Rahnenfuhrer, 2021). Statistical enrichment of GO molecular function and GO biological process terms was performed between children conceived spontaneously, following IVF and ICSI-TESE using the limma and edgeR packages (Ritchie *et al.*, 2015; Chen *et al.*, 2016). The analysis was also performed between groups of children born to younger and older fathers.

## Results

The main hypothesis tested in this pilot study was that the germline genomes of children born after MAR contain more DNMs than those born through spontaneous conception, independently of parental age at conception. The number of DNMs present in the genome of children conceived spontaneously and conceived via an ART were evaluated by performing WGS on 53 children and their respective parents using BGIseq-500 at an average coverage depth of 38× (Fig. 1, Supplementary Tables SI and SII). Replicate WGS was performed for 34 trios on the same platform. The detection of DNMs is strongly dependent on the quality of the genome sequencing approach used, and we extensively evaluated and optimized our DNM detection method using these replicate WGS datasets. After filtering for high confidence DNMs, 95% of all predicted DNMs from WGS run 1 were also identified as predicted DNMs in WGS run 2 (Supplementary Tables SIII and SIV). We further confirmed the specificity of our DNM calling by performing Sanger sequencing on 81 randomly selected predicted DNMs from all groups, validating 80 as DNM and 1 as inherited (99% validation rate) (Supplementary Table SV). In total, 4344 high confidence DNMs were identified across all groups, with 98% of the mutations occurring outside the coding region of the genome (Supplementary Table SIV).

**Figure 2.**
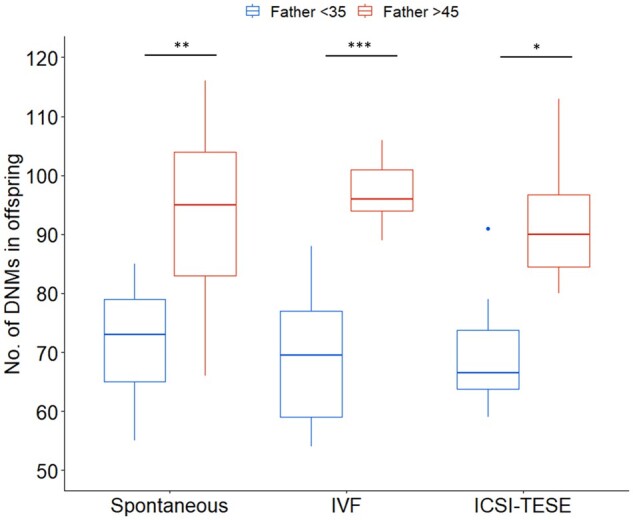
**Number of *de novo* mutations (DNMs) in the genome of the children conceived by different methods and paternal age at the time of conception.** Boxplot represents the distribution of DNMs observed in genome of children displaying minimum, first quartile, median, third quartile and maximum number of DNMs in children per group, in blue for children born to fathers younger than 35 years of age at the time of conception (<35) and in red for children born to fathers older than 45 years of age at conception (>45). Outliers are represented as a single dot on the plot. Multi-factorial ANOVA detected no significant differences in the number of DNMs in children conceived spontaneously or via MAR; however, it detected a significant association between the number of DNMs and the age of the fathers at conception which a *post hoc* Tukey test revealed to be significant within each group. ICSI-TESE, ICSI combined with testicular sperm extraction; *P-value < 0.05; **P-value < 0.01; ***P-value < 0.001.

**Figure 3.**
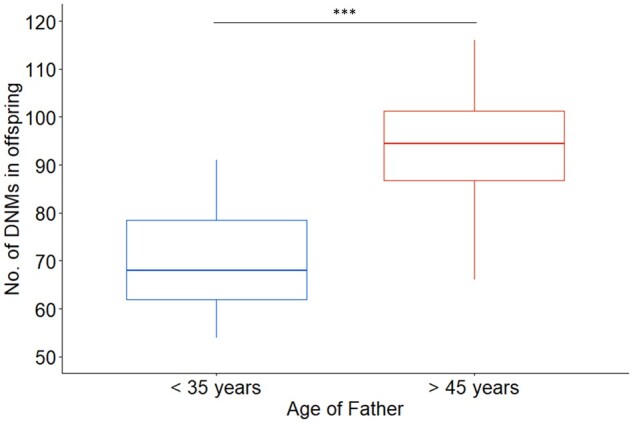
**Number of *de novo* mutations (DNMs) in relation to father’s age at time of conception.** Boxplot represents the distribution of DNMs observed in genome of children displaying minimum, first quartile, median, third quartile and maximum number of DNMs in children per group, in blue for children born to fathers younger than 35 years of age at the time of conception (<35) and in red for children born to fathers older than 45 years of age at conception (>45). Independently of the method of conception there is a highly statistically significant difference in the number of DNMs in the genome of children born from young fathers and those born from older fathers (independent Student’s *t*-test, *t*(50) = −7.9, *P*-value = 6.89 × 10^−10^). ICSI-TESE, ICSI combined with testicular sperm extraction; ***P-value < 0.001.

**Figure 4.**
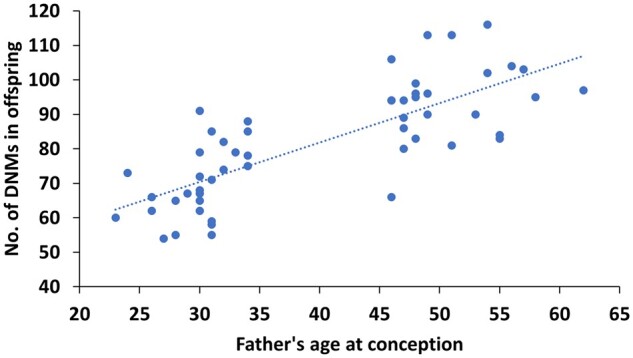
**Relationship between the father’s age at conception and the number of *de novo* mutations (DNMs) present in the genome of the offspring.** Dotted line visually represents the goodness-of-fit measure for multiple regression model (*R*^2^ = 0.6113) for correlation between father’s age and DNMs in their respective children.

Overall, we found no significant difference in the number of DNMs in the children conceived naturally or via MAR (multi-factorial ANOVA, *f*(2) = 0.17, *P*-value = 0.85) nor a significant interaction between parental age and the method of conception influencing the number of DNMs found in these children (multi-factorial ANOVA, *f*(2) = 0.66, *P*-value = 0.50). Separately, maternal and paternal age at the time of conception was found to have no significant interaction with the method of conception to influence the number of the DNMs in children (multi-factorial ANOVA, *F*(2) = 1.85, *P*-value = 0.17 and *f*(2) = 1.85, *P*-value = 0.13, respectively). However, as expected paternal age at conception was significantly associated with the number of DNMs in the offspring, independently of the method of conception and maternal age (multi-factorial ANOVA, *f*(2) = 82.76, *P*-value = 2.19 × 10^−11^) (Table I, Fig. 2). This was also observed when comparing the DNM number in children born to older and younger fathers, independently of any other factor (independent Student’s *t*-test, *t*(50) = −7.9, *P*-value = 6.89 × 10^−10^) (Fig. 3). In the control group of spontaneous conception, children born to younger fathers were found to have on average 71 DNMs in their genome, compared to 94 DNMs in children born to older fathers (*post hoc* Tukey test, adjusted *P*-value = 0.001) (Fig. 2). In the group of children born from younger fathers, the control group of spontaneous conception had on average 71 ± 9 DNMs in their genome, compared to 69 ± 15 DNMs in the IVF group (*post hoc* Tukey test, adjusted *P*-value = 1.0) and 70 ± 11 DNMs in the ICSI-TESE group (*post hoc* Tukey test, adjusted *P*-value = 1.0). In the group of children born from older fathers, the control group of spontaneous conception were on average 94 ± 5 DNMs in their genome, compared to 97 ± 10 and 92 ± 10 DNM in the IVF and ICSI-TESE respectively (*post hoc* Tukey test, adjusted *P*-value = 1.0 both). By applying a multiple regression model to investigate the impact of the method of conception, age of father and age of mother at the time of conception, it was determined that neither the method of conception (multiple regression model, *t* = 0.396, *P*-value = 0.694) nor the age of the mother (multiple regression model, *t* = 0.396, *P*-value = 0.639) had a significant effect on the number of DNMs in the children. Again, the age of the father at conception was found to have a significant effect (multiple regression model, *t* = 5.636, *P*-value = 8.97 × 10^−7^) on the number of DNMs present in the genome of their offspring, independently from the method of conception, with an estimated increase of 1.09 ± 0.19 DNMs per year increase with father’s age (multiple regression model, *F*(4,48) = 18.87, *R*2 = 0.6113, *P*-value = 2.22 × 10^−9^, Fig. 4). In total, across all children in this study, 82 high confidence DNMs were found to affect the coding regions of the genome, of which 22 were identified to cause a synonymous mutation and thus unlikely to affect the encoded protein (Supplementary Table SIV). Following GO term annotation for each of the genes affected by a protein altering DNM, an enrichment analysis was performed to investigate whether specific molecular mechanisms or biological processes were consistently affected. However, no statistical enrichment was detected in GO terms between the groups of children conceived naturally and via MAR or between groups of children born to younger and older fathers.

**Table I:** Distribution of number of *de novo* mutations across the different treatment groups.

Children ID number	Method of conception	No. of DNMs in children	Maternal age at conception	Paternal age at conception	Fertility status
**101C**	Spontaneous	85	32	34	No fertility problem
**102C**	Spontaneous	65	28	28	No fertility problem
**103C**	Spontaneous	79	33	33	No fertility problem
**105C**	Spontaneous	62	27	26	No fertility problem
**106C**	Spontaneous	82	37	32	No fertility problem
**107C**	Spontaneous	75	29	34	No fertility problem
**108C**	Spontaneous	73	23	24	No fertility problem
**112C**	Spontaneous	67	32	30	Severe oligozoospermia, pregnant while waiting for treatment
**114C**	Spontaneous	55	30	31	Ovulation disorder, pregnant while waiting for treatment

**Group average**	**Spontaneous <35**	**71**	**30**	**30**	

**201C**	Spontaneous	95	40	58	No fertility problem
**202C**	Spontaneous	116	28	54	No fertility problem
**205C**	Spontaneous	95	41	48	No fertility problem
**206C**	Spontaneous	81	42	51	Severe oligozoospermia, pregnant while waiting for treatment
**207C**	Spontaneous	104	37	56	Unexplained infertility, pregnant while waiting for treatment
**209C**	Spontaneous	113	37	49	No fertility problem
**210C**	Spontaneous	94	38	46	No fertility problem
**211C**	Spontaneous	83	39	48	Unexplained infertility, pregnant while waiting for treatment
**212C**	Spontaneous	66	35	46	Moderate oligozoospermia, pregnant while waiting for treatment

**Group average**	**Spontaneous >45**	**94**	**37**	**51**	

**302C**	IVF	54	30	27	Endometriosis
**303C**	IVF	55	28	28	Endometriosis
**304C**	IVF	68	28	30	Endometriosis
**305C**	IVF	58	30	31	Unexplained infertility
**307C**	IVF	74	32	32	Unexplained infertility
**308C**	IVF	62	27	30	Unexplained infertility
**310C**	IVF	71	27	31	Endometriosis
**311C-1**	IVF	85	32	31	Unexplained infertility
**311C-2**	IVF	88	35	34	Unexplained infertility
**312C**	IVF	78	33	34	Unexplained infertility

**Group average**	**IVF <35**	**69**	**30**	**31**	

**402C**	IVF	103	37	57	Unexplained infertility
**403C**	IVF	89	38	47	Unexplained infertility
**404C**	IVF	96	41	48	Unexplained infertility
**408C**	IVF	106	41	46	Tubal pathology
**409C**	IVF	94	29	46	Cervical factor
**410C**	IVF	99	36	48	Unexplained infertility
**414C**	IVF	94	37	47	Tubal pathology
**Group average**	**IVF >45**	**97**	**37**	**48**	

**503C**	ICSI-TESE	60	30	23	NOA
**505C**	ICSI-TESE	79	33	30	Extreme oligoasthenoteratozoospermia
**506C**	ICSI-TESE	72	30	30	OA based on CBAVD
**507C**	ICSI-TESE	65	26	30	OA based on CBAVD
**508C**	ICSI-TESE	91	25	30	NOA
**509C**	ICSI-TESE	67	27	29	NOA
**510C**	ICSI-TESE	66	23	26	NOA
**511C**	ICSI-TESE	59	26	31	NOA

**Group average**	**ICSI-TESE <35**	**70**	**28**	**29**	

**601C**	ICSI-TESE	80	27	47	OA after sterilisation: PESA no sperm was found
**606C**	ICSI-TESE	102	40	54	OA after vasovasostomy
**607C**	ICSI-TESE	86	25	47	OA after vasovasostomy
**609C**	ICSI-TESE	113	38	51	OA after sterilisation: with PESA no sperm was found
**610C**	ICSI-TESE	97	35	62	OA after sterilisation: with PESA no sperm was found
**611C**	ICSI-TESE	96	31	49	OA after sterilisation: with PESA no sperm was found
**612C**	ICSI-TESE	90	33	49	OA after vasovasostomy
**613C**	ICSI-TESE	90	39	53	OA after sterilisation: with PESA no sperm was found
**614C-1**	ICSI-TESE	84	38	55	OA after vasovasostomy
**614C-2**	ICSI-TESE	83	38	55	OA after vasovasostomy

**Group average**	**ICSI-TESE >45**	**92**	**34**	**52**	

<35, children born to fathers younger than 35 years of age at time of conception; >45, children born to fathers older than 45 years of age at time of conception; CBAVD, congenital bilateral absence of the vas deferens; DNMs, *de novo* mutations; ICSI-TESE, ICSI combined with testicular sperm extraction; NOA, non-obstructive azoospermia; OA, obstructive azoospermia; PESA, percutaneous epididymal sperm aspiration.

Phasing analysis and identification of parent-of-origin were performed for all autosomal DNMs across all methods of conception being investigated. In total, 1173 out of the 4200 identified autosomal DNMs (28%) could be phased and have parental origin assigned to. Overall, 75% of all DNM were of paternal origin (varying between 72% and 78% in the different subgroups). No significant difference was observed in the parent-of-origin of the DNMs present in the genomes of children conceived spontaneous or via MAR adjusting for paternal and maternal age at conception (negative binomial regression, *z*(43) = −0.22, *P*-value = 0.8) (Supplementary Table SVI).

A more in-depth analysis showed that 3% of all DNMs occur within 20 kb distance of each other and can be classified as being part of a cluster of DNMs. In total, we identified 62 DNM clusters with on average 2.2 DNMs per cluster (Supplementary Tables SVII and SVIII). For 93% of all clusters, the DNMs were found to occur at the same parental allele, indicating a single genomic mutational event (Supplementary Table SIX). No differences were observed between the number of DNM clusters for the different methods of conception adjusting for parental age at conception (multi-factorial ANOVA, *F*(2) = 0.14, *P*-value = 0.87).

## Discussion

This pilot study suggests that there is no significant relation between the method of conception and the number of DNMs in the genomes of offspring. In other words, children born with MAR do not seem to carry more DNMs in their genome than children born after spontaneous conception.

The high quality of our DNM detection is corroborated by our replicated genome sequencing of 34 of our trios which showed that 95% of all predicted DNMs were identified in both experiments. The strength of our methodology was further confirmed by independent validation of 80 out of 81 randomly selected DNMs as real DNMs by Sanger sequencing (99% validation rate). In addition, the number of DNM detected in the genome of these children (54–116 DNMs per genome), is consistent with the previously reported rate of 44 to 89 DNMs per genome in the human population (Kong *et al.*, 2012; Francioli *et al.*, 2015; Acuna-Hidalgo *et al.*, 2016; Goldmann *et al.*, 2016). Recently, DNA extracted from cord blood cells and placental tissue has been used to investigate the number of *de novo* copy number variations (CNVs) present in the genome of new-borns conceived with IVF and in spontaneously conceived new-borns (Zamani Esteki *et al.*, 2019). In line with our findings, these authors did not see a detrimental effect of IVF on the amount of *de novo* genomic aberrations or large structural DNA imbalances.

Our study does show a clear paternal age effect on the number of DNMs in the offspring, with an estimate effect of 1.1 DNMs per year increase in the father’s age at conception. Kong et al. were the first to report on this paternal age effect (Kong *et al.*, 2012). This paternal age effect has been shown to have a negative effect on the health and well-being of the offspring by increasing the occurrence of disease in children born to older fathers (Hultman *et al.*, 2011; Taylor *et al.*, 2019). Additionally, there seems to be a clear link between men’s age and the deterioration of sperm DNA quality, suspected to lead to an increased mutational load in offspring (Aitken *et al.*, 2020; Evenson *et al.*, 2020). Furthermore, a recent study on 1500 parent-offspring trios showed evidence that germline variants not only arise from replication errors in germ cells, but that DNA damage-induced mutations might also play a substantial role (Gao *et al.*, 2019). In particular, damage caused by radiation and chemotherapy, often leading to transient or permanent azoospermia (Howell, 2005; Meistrich, 2013) in patients that then require MAR to successfully conceive, has also been shown to cause multi-site DNMs and germline hypermutation, respectively (Holtgrewe *et al.*, 2018; Kaplanis *et al.*, 2021).

In conjunction with the paternal age effect influencing the number of DNMs in the genome of children, phasing analysis on 1220 out of all DNMs identified that on average 75% of DNMs were of paternal origin, in line with the 80% reported in literature (Crow, 2000; Kong *et al.*, 2012; Goldmann *et al.*, 2016; Yuen *et al.*, 2016). Similar parent-of-origin results were obtained for DNMs present in the genomes of children conceived spontaneously or via MAR. Furthermore, 2.2% of our DNMs occurred in close proximity to each other, similar to the 2.5% of clustered DNMs previously reported (Goldmann *et al.*, 2016, 2019). In accordance with the literature (Yuen *et al.*, 2016; Goldmann *et al.*, 2018), clusters of DNM events showed a greater maternal bias rather than paternal, independently of the method of conception. These findings are in line with previous reports showing that clusters of DNMs are caused by the deficient homologous-recombination repair of double-strand breaks due to an ageing oocyte’s DNA repair mechanism. This allows the creation of deregulated recombination hotspots and mutations to occur closer to each other than otherwise expected (Campbell *et al.*, 2015; Yuen *et al.*, 2016; Goldmann *et al.*, 2018). Importantly, however, again no differences were observed between the number of DNM clusters for the different methods of conception.

In contrast to our findings, Wong *et al.* (2016) reported a small increase in the number of DNMs per genome for 25 children born after MAR. Unfortunately, the authors did not provide any information regarding the ART method used, the age of the parents at the time of conception nor the reason for couples to undergo MAR, so it is difficult for us to comment on this observed difference. A recently reported study by Wang *et al.* (2021) also suggests that children conceived through MAR carry more DNMs in their genome compared to children conceived spontaneously. While there is no clear explanation for the discrepancy between this study and ours, we did note that the total number of DNMs observed per genome per offspring deviates significantly between our study and that of Wang *et al.* They identified an average of 39 DNMs per offspring, while we identify an average of 82 DNMs, with high accuracy as discussed above. Our data also seem to be more in line with the reported number of DNMs per genome, roughly varying between 44 and 89 DNMs (Kong *et al.*, 2012; Francioli *et al.*, 2015; Acuna-Hidalgo *et al.*, 2016; Goldmann *et al.*, 2016). Differences in paternal age at conception, which significantly affect the number of DNMs per offspring, could explain why we identify more DNMs. However, even in our sub-cohort of children born to fathers younger than 35 years, we identified an average of 71 DNMs, significantly more than observed by Wang *et al.* In addition, they focus their statistical analysis on DNMs which could be phased to either one of the parents, leaving a very low number of DNMs per offspring for the statistical association with methods of conception. Larger follow-up studies will clearly be required to resolve the discrepancies reported. Our pilot study reliably identified well-known large effects on the DNM rate such as the paternal age effect but did not identify more subtle effects such as the maternal age effect (Goldmann *et al.*, 2016). We can therefore exclude that the method of conception has a similarly large effect as that of paternal age at conception. To detect more subtle effects, we would need at least 54 individuals in each paternal age group (80% power, 0.05 significance level).

In conclusion, our pilot study showed no impact of assisted reproductive technologies on the number of DNMs in the genome of the offspring. Given the role DNMs play in disease risk, this negative result is potentially good news for IVF and ICSI-TESE born children, if replicated in a larger cohort. These findings highlight the continued need to investigate the consequences of MAR particularly now that so many couples are being counselled to consider these techniques to overcome their fertility issues. While in this study we focused on the effect of MAR on the number of germline DNMs, it may also be worthwhile to look at the effect on the number of somatic DNMs present in the offspring or the number of CNVs. Overall, our data provide important insight into possible genetic risks associated with the use of specific ART methods and MAR in general.

## Supplementary data

Supplementary data are available at *Human Reproduction* online.

## Data availability 

The data underlying this article are available under controlled access from the European Genome-Phenome Archive (EGA) at https://ega-archive.org/ and can be accessed with the unique study identifier EGAS00001005569.

## Supplementary Material

deac068_Supplementary_Table_SI

deac068_Supplementary_Table_SII

deac068_Supplementary_Table_SIII

deac068_Supplementary_Table_SIV

deac068_Supplementary_Table_SV

deac068_Supplementary_Table_SVI

deac068_Supplementary_Table_SVII

deac068_Supplementary_Table_SVIII

deac068_Supplementary_Table_SIX
