# Supplementary material for: De novo mutations in children born after medical assisted reproduction
Source: Hum Reprod. 2022 Apr 12;37(6):1360–9. doi: 10.1093/humrep/deac068 (PMC9156847; doi:10.1093/humrep/deac068)
Supplement: deac068_Supplementary_Table_SII [file deac068_supplementary_table_sii.pdf]

Human Reproduction, pp. 1–2, 2022
https://doi.org/10.1093/humrep/deac068

human reproduction

SUPPLEMENTARY DATA

Supplementary Table SII Mean depth of coverage for WGS samples included in study.

Children ID number	Group	Patient mean coverage	Maternal mean coverage	Paternal mean coverage	Additional sequencing		
					Patient mean coverage	Maternal mean coverage	Paternal mean coverage
101C	Spontaneous <35	36X	37X	29X	38X	44X	39X
102C	Spontaneous <35	42X	32X	34X	41X	45X	40X
103C	Spontaneous <35	31X	39X	40X	37X	33X	36X
105C	Spontaneous <35	32X	33X	32X	40X	38X	35X
106C	Spontaneous <35	37X	39X	28X	47X	42X	43X
107C	Spontaneous <35	39X	44X	30X	—	—	—
108C	Spontaneous <35	38X	39X	29X	50X	37X	37X
112C	Spontaneous <35	38X	35X	36X	—	—	—
114C	Spontaneous <35	29X	35X	39X	—	—	—
201C	Spontaneous >45	32X	30X	37X	36X	45X	38X
202C	Spontaneous >45	39X	40X	37X	31X	41X	39X
205C	Spontaneous >45	31X	36X	42X	—	—	—
206C	Spontaneous >45	39X	48X	48X	—	—	—
207C	Spontaneous >45	30X	29X	30X	40X	41X	41X
209C	Spontaneous >45	34X	34X	30X	40X	41X	40X
210C	Spontaneous >45	33X	45X	38X	33X	45X	38X
211C	Spontaneous >45	38X	45X	41X	—	—	—
212C	Spontaneous >45	32X	41X	41X	32X	41X	41X
302C	IVF <35	41X	35X	30X	40X	44X	44X
303C	IVF <35	36X	38X	31X	34X	40X	29X
304C	IVF <35	47X	33X	41X	32X	44X	34X
305C	IVF <35	31X	28X	36X	46X	26X	30X
307C	IVF <35	36X	45X	35X	—	—	—
308C	IVF <35	29X	31X	31X	39X	38X	40X
310C	IVF <35	31X	38X	47X	—	—	—
311C-1	IVF <35	43X	40X	36X	—	—	—
311C-2	IVF <35	44X	40X	36X	—	—	—
312C	IVF <35	44X	48X	42X	—	—	—
402C	IVF >45	33X	39X	32X	—	—	—
403C	IVF >45	37X	34X	28X	29X	24X	29X
404C	IVF >45	32X	35X	43X	34X	23X	33X
408C	IVF >45	42X	51X	33X	39X	36X	31X
409C	IVF >45	33X	36X	42X	34X	34X	40X
410C	IVF >45	35X	38X	38X	45X	42X	45X
414C	IVF >45	48X	47X	36X	—	—	—
503C	ICSI-TESE <35	42X	45X	30X	37X	37X	40X
505C	ICSI-TESE <35	39X	50X	38X	37X	29X	26X
506C	ICSI-TESE <35	29X	37X	39X	30X	30X	25X
507C	ICSI-TESE <35	38X	28X	33X	33X	27X	11X
508C	ICSI-TESE <35	41X	39X	45X	—	—	—
509C	ICSI-TESE <35	39X	46X	48X	29X	31X	23X

(continued)

Supplementary Table SII Continued

Children ID number	Group	Patient mean coverage	Maternal mean coverage	Paternal mean coverage	Additional sequencing		
					Patient mean coverage	Maternal mean coverage	Paternal mean coverage
510C	ICSI-TESE <35	57X	34X	49X	38X	35X	31X
511C	ICSI-TESE <35	29X	35X	28X	39X	35X	38X
601C	ICSI-TESE >45	39X	27X	42X	—	—	—
606C	ICSI-TESE >45	33X	41X	42X	33X	33X	23X
607C	ICSI-TESE >45	31X	32X	41X	42X	45X	38X
609C	ICSI-TESE >45	39X	35X	37X	—	—	—
610C	ICSI-TESE >45	30X	30X	41X	33X	41X	38X
611C	ICSI-TESE >45	49X	46X	42X	33X	40X	39X
612C	ICSI-TESE >45	33X	35X	29X	—	—	—
613C	ICSI-TESE >45	58X	46X	30X	34X	37X	37X
614C-1	ICSI-TESE >45	29X	54X	46X	—	—	—
614C-2	ICSI-TESE >45	31X	54X	46X	—	—	—
**Average coverage:**		**37X**	**39X**	**37X**	**37X**	**37X**	**35X**

<35, children born to fathers younger than 35 years of age at time of conception; >45, children born to fathers older than 45 years of age at time of conception; DNMs, *de novo* mutations; ICSI-TESE, ICSI combined with testicular sperm extraction.
