# Supplementary material for: De novo mutations in children born after medical assisted reproduction
Source: Hum Reprod. 2022 Apr 12;37(6):1360–9. doi: 10.1093/humrep/deac068 (PMC9156847; doi:10.1093/humrep/deac068)
Supplement: deac068_Supplementary_Table_SIII [file deac068_supplementary_table_siii.pdf]

Human Reproduction, pp. 1–2, 2022
https://doi.org/10.1093/humrep/deac068

human reproduction

SUPPLEMENTARY DATA

Supplementary Table SIII High confidence DNM calling from replicate WGS of selected trios.

Children ID number	Group	Total high confidence DNMs	Number of high confidence mutations confirmed by replicate genome sequencing	Number of high confidence mutations not replicated	Percentage of DNMs present in replicate genome sequencing
101C	Spontaneous <35	85	76	9	89%
102C	Spontaneous <35	65	63	2	97%
103C	Spontaneous <35	79	77	2	97%
105C	Spontaneous <35	62	57	5	92%
106C	Spontaneous <35	82	78	4	95%
107C	Spontaneous <35	75	—	—	—
108C	Spontaneous <35	73	70	3	96%
112C	Spontaneous <35	67	—	—	—
114C	Spontaneous <35	55	—	—	—
201C	Spontaneous >45	95	93	2	98%
202C	Spontaneous >45	116	105	11	91%
205C	Spontaneous >45	95	—	—	—
206C	Spontaneous >45	81	—	—	—
207C	Spontaneous >45	104	101	3	97%
209C	Spontaneous >45	113	111	2	98%
210C	Spontaneous >45	94	92	2	98%
211C	Spontaneous >45	83	—	—	—
212C	Spontaneous >45	66	64	2	97%
302C	IVF <35	54	53	1	98%
303C	IVF <35	55	53	2	96%
304C	IVF <35	68	66	2	97%
305C	IVF <35	58	55	3	95%
307C	IVF <35	74	—	—	—
308C	IVF <35	62	60	2	97%
310C	IVF <35	71	—	—	—
311C-1	IVF <35	85	—	—	—
311C-2	IVF <35	88	—	—	—
312C	IVF <35	78	—	—	—
402C	IVF >45	103	—	—	—
403C	IVF >45	89	84	5	94%
404C	IVF >45	96	93	3	97%
408C	IVF >45	106	103	3	97%
409C	IVF >45	94	87	7	93%
410C	IVF >45	99	99	0	100%
414C	IVF >45	94	—	—	—
503C	ICSI-TESE <35	60	58	2	97%
505C	ICSI-TESE <35	79	75	4	95%
506C	ICSI-TESE <35	72	66	6	92%
507C	ICSI-TESE <35	65	54	11	83%
508C	ICSI-TESE <35	91	—	—	—
509C	ICSI-TESE <35	67	60	7	90%
510C	ICSI-TESE <35	66	62	4	94%

(continued)

Supplementary Table SIII Continued

Children ID number	Group	Total high confidence DNMs	Number of high confidence mutations confirmed by replicate genome sequencing	Number of high confidence mutations not replicated	Percentage of DNMs present in replicate genome sequencing
511C	ICSI-TESE <35	59	56	3	95%
601C	ICSI-TESE >45	80	—	—	—
606C	ICSI-TESE >45	102	95	7	93%
607C	ICSI-TESE >45	86	80	6	93%
609C	ICSI-TESE >45	113	—	—	—
610C	ICSI-TESE >45	97	92	5	95%
611C	ICSI-TESE >45	96	91	5	95%
612C	ICSI-TESE >45	90	—	—	—
613C	ICSI-TESE >45	90	86	4	96%
614C-1	ICSI-TESE >45	84	—	—	—
614C-2	ICSI-TESE >45	83	—	—	—

<35, children born to fathers younger than 35 years of age at time of conception; >45, children born to fathers older than 45 years of age at time of conception; DNMs, *de novo* mutations; ICSI-TESE, ICSI combined with testicular sperm extraction.
