# Supplementary material for: De novo mutations in children born after medical assisted reproduction
Source: Hum Reprod. 2022 Apr 12;37(6):1360–9. doi: 10.1093/humrep/deac068 (PMC9156847; doi:10.1093/humrep/deac068)
Supplement: deac068_Supplementary_Table_SVI [file deac068_supplementary_table_svi.pdf]

Human Reproduction, pp. 1–1, 2022
https://doi.org/10.1093/humrep/deac068

human reproduction

SUPPLEMENTARY DATA

Supplementary Table SVI Number of *de novo* mutations (DNMs) used in phasing analysis to determine parent-of-origin across spontaneous and or via medical assisted reproduction (MAR).

Group	Total no. of DNMs	Phasable DNMs	Percentage of phasable DNMs (%)	DNMs paternal in origin (%)	DNMs maternal in origin (%)
Spontaneous <35	643	200	31.1	77.0	23.0
Spontaneous >45	847	267	31.5	76.0	24.0
IVF <35	693	114	16.5	75.0	25.0
IVF >45	681	222	32.6	72.2	27.8
ICSI-TESE <35	559	138	24.7	74.0	26.0
ICSI-TESE >45	921	232	25.2	78.0	22.0

<35, children born to fathers younger than 35 years of age at time of conception; >45, children born to fathers older than 45 years of age at time of conception; DNMs, *de novo* mutations; ICSI-TESE, ICSI combined with testicular sperm extraction.
