# Supplementary material for: De novo mutations in children born after medical assisted reproduction
Source: Hum Reprod. 2022 Apr 12;37(6):1360–9. doi: 10.1093/humrep/deac068 (PMC9156847; doi:10.1093/humrep/deac068)
Supplement: deac068_Supplementary_Table_SVII [file deac068_supplementary_table_svii.pdf]

Human Reproduction, pp. 1–1, 2022
https://doi.org/10.1093/humrep/deac068

human reproduction

SUPPLEMENTARY DATA

Supplementary Table SVII ***De novo*** **mutation (DNM) clusters found in the genomes of children born spontaneously and conceived or via medical assisted reproduction (MAR).**

Group	Total no. of DNMs	No. of DNM clusters	No. of DNMs in clusters	Percentage of DNMs in clusters (%)	Average No. of DNMs per cluster
Spontaneous <35	643	6	12	1.9	2.0
Spontaneous >45	847	15	32	3.8	2.1
IVF <35	693	7	16	2.3	2.3
IVF >45	681	11	24	3.5	2.2
ICSI-TESE <35	559	7	14	2.5	2.0
ICSI-TESE >45	921	16	40	4.3	2.5

<35, children born to fathers younger than 35 years of age at time of conception; >45, children born to fathers older than 45 years of age at time of conception; DNMs, *de novo* mutations; ICSI-TESE, ICSI combined with testicular sperm extraction.
